# Nanotribology at high temperatures

**DOI:** 10.3762/bjnano.3.68

**Published:** 2012-08-15

**Authors:** Saurav Goel, Alexander Stukowski, Gaurav Goel, Xichun Luo, Robert L Reuben

**Affiliations:** 1 School of Engineering and Physical Sciences, Heriot-Watt University, Edinburgh, EH144AS, UK; 2 School of Computing and Engineering, University of Huddersfield, Huddersfield, HD13DH, UK; 3 Lawrence Livermore National Laboratory, Livermore, California, USA; 4 School of Engineering and Technology, Sharda University, Greater Noida, 201306, India

**Keywords:** CBN, diamond, high temperature

## Abstract

Recent molecular dynamics simulation results have increased conceptual understanding of the grazing and the ploughing friction at elevated temperatures, particularly near the substrate’s melting point. In this commentary we address a major constraint concerning its experimental verification.

## Introduction

It was postulated some time ago that a component sliding under lightly loaded conditions should experience very low friction and nearly zero wear [1]. Recent research, however, has shown a steep rise in the grazing friction during wearless sliding, primarily attributed to the adhesion between the interacting surfaces [2]. A major assumption in the atomistic simulation associated with this finding was the consideration of the diamond tip as a wearless rigid body for ease of computation. Accordingly, it has been suggested [2] that the steep rise in grazing friction and the gradual drop in ploughing friction at high temperatures may affect all materials and should be pursued experimentally [3].

However, diamond is known to exhibit poor thermo-chemo-mechanical stability particularly against low carbon ferrous alloys [4] and at elevated temperatures [5]. It is surprising, but true, that diamond, the hardest material available (until the commercial realization of beta carbon nitride β-(C_3_N_4_) [6]), wears out rapidly when it is rubbed against low carbon ferrous alloys and pure iron [7]. A hypothesis was proposed by Paul et al. [8] ascribing the rapid chemical wear of diamond tips to the presence of unpaired d-shell electrons in the substrate. Utilizing this proposition, a research group from Bremen University, Germany, has reversed the roles of the conventionally used cutting tool and the workpiece material and has succeeded in machining a diamond substrate using a steel rod as a tip [9].

This raises a key question concerning the stability of diamond tips during nanoscratch tests at elevated temperatures and more categorically against low carbon ferrous alloys. The wear of the diamond tip will change the contact area which will alter the frictional force as the latter depends linearly on the number of atoms that chemically interact across the surface [10]. Then, the question arises as to what other options are available for a tool tip for nanotribological applications at elevated temperatures, if not diamond. We attempt to answer this question below.

## Discussion

**Figure 1:**
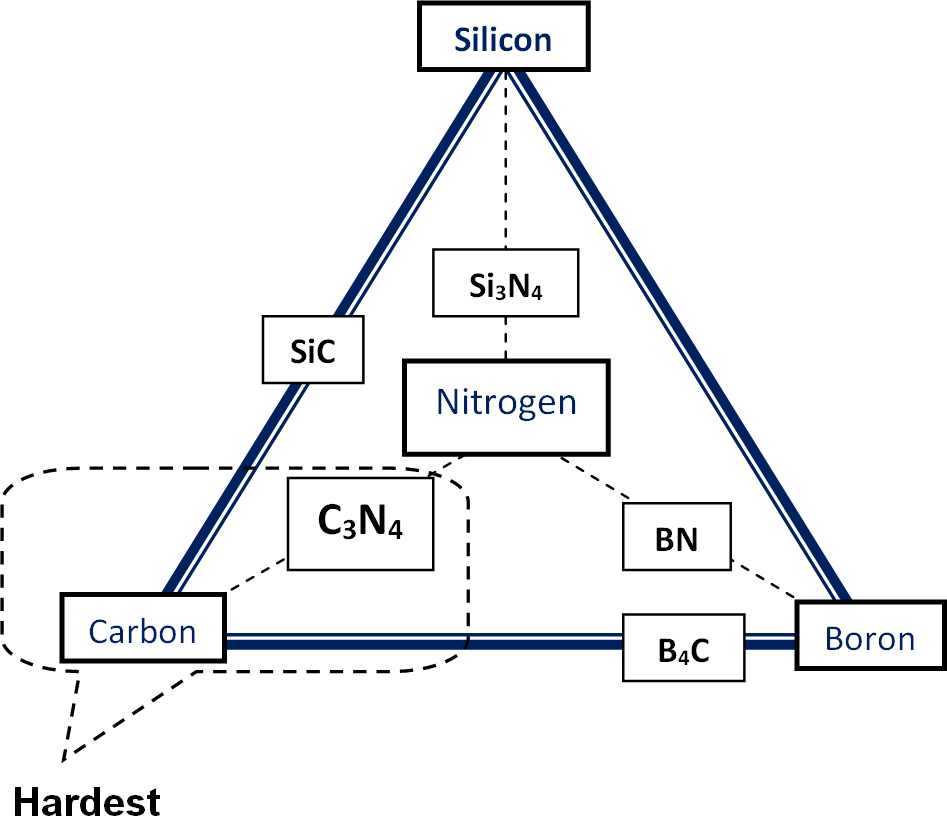
Composition cycle of ultra-hard materials (C–N–B–Si) [11].

Principally, ultra-hard materials – materials whose hardness is attributable to covalent bonding – can be represented using the “composition cycle” shown in Figure 1. This cycle involves the four elements carbon (C), boron (B), silicon (Si) and nitrogen (N). The combination of any two chemical species from this composition cycle produces a compound exhibiting ultra-high hardness, e.g., CBN, SiC, Si_3_N_4_, B_4_C and the recently recognized C_3_N_4_.

Under ambient conditions – while nitrogen reside in a gaseous form as individual chemical species – boron, silicon and diamond are known to prefer a solid state. Due to its abundance and its capability to form better oxides, silicon dominated the electronic consumer market [12] while the ubiquitous use of diamond originates from its unique features such as high thermal conductivity, high wear resistance and its ability to form extremely sharp cutting edges [13]. Moreover, both diamond and silicon are known to be hard and brittle [14–15] in their sp^3^-bonded state. Two commercially available materials from this composition cycle, diamond (C) and cubic boron nitride (CBN), possess ultra-high hardness (attributed both to sp^3^-bonding and relatively short bond lengths) and, for this reason, they are frequently used to manufacture cutting tips. While diamond resides in a cubic lattice structure, CBN possesses a zinc-blende structure having boron atoms at the crystal site (0,0,0) and nitrogen atoms in the respective centers of the boron tetrahedra. Although, a chemical bond between the carbon atoms in diamond is stronger than the corresponding isoelectronic bond between nitrogen and boron atoms, we propose that cubic boron nitride “CBN” could be an alternative appropriate choice for high-temperature nanotribology applications because of its superior thermal and chemical stability compared to that of diamond. Even though diamond and CBN have similar lattice structures, their surface chemistry is different. In a CBN lattice, boron atoms have only three valence electrons on the surface while nitrogen atoms have five. However, two of these five electrons of nitrogen can form a stable pair, leaving three valence electrons to bond with boron. On the contrary, a diamond lattice has four valence electrons; therefore, only a maximum of three electrons on the surface can have stable bonding between them. Consequently, this leads to the possibility that the remaining one or two electrons of each surface atom in diamond react readily with other materials like iron, nickel and even silicon [16] in a tribological environment. This seems to be a plausible reason why CBN was found as an efficient cutting tip to machine ferrous alloys [17] and silicon [18] in comparison to a diamond tip. Hence, in contrast to diamond, CBN has fewer dangling bonds on the surface which makes it more inert.

## Conclusion

It is proposed that the results for the ploughing friction and grazing friction in a high temperature environment must be retested using a CBN probe to reconfirm the state-of-the-art knowledge. Moreover, the influence of the rake angle of the tip must necessarily be accounted for when generalizing the results of grazing friction, as it is an integral part in governing atomic scale friction [19–20].
